# Virtual Reality as a Therapy Tool for Walking Activities in Pediatric Neurorehabilitation: Usability and User Experience Evaluation

**DOI:** 10.2196/38509

**Published:** 2022-07-14

**Authors:** Corinne Ammann-Reiffer, Andrina Kläy, Urs Keller

**Affiliations:** 1 Research Department Swiss Children's Rehab University Children's Hospital Zurich - Eleonore Foundation Affoltern am Albis Switzerland; 2 Children’s Research Center University Children’s Hospital Zurich - Eleonore Foundation Zurich Switzerland

**Keywords:** rehabilitation, pediatric, child, adolescent, walking, feasibility study, virtual reality, head-mounted display, therapy, tool, user, usability, walking, visual, auditory, feedback, youth

## Abstract

**Background:**

Many essential walking activities in daily life, such as crossing a street, are challenging to practice in conventional therapeutic settings. Virtual environments (VEs) delivered through a virtual reality (VR) head-mounted display (HMD) would allow training such activities in a safe and attractive environment. Furthermore, the game-like character and high degree of immersion in these applications might help maintain or increase children’s motivation and active participation during the rehabilitation process.

**Objective:**

This study aimed to investigate the usability, user experience, and acceptability of an immersive VE experienced through a VR HMD to train everyday life walking activities in pediatric neurorehabilitation.

**Methods:**

In a cross-sectional study, 21 youths (median age 12.1 years; range 6.8-17.7 years) with a neuromotor impairment undergoing inpatient or outpatient neurorehabilitation tested a VE experienced through the VR HMD Oculus Quest. The participants, accompanied by their physiotherapists, moved freely around a 4.4 by 10-meter VE, displaying a magical forest and featuring various gamified everyday activities in different game designs. Using their hands, represented in the VE, the participants could interact with the virtual objects placed throughout the VE and trigger visual and auditory feedback. Symptoms of cybersickness were checked, and usability, user experience, and acceptability were evaluated using customized questionnaires with a visual analog scale for youths and a 5-point Likert scale for their therapists.

**Results:**

None of the participants reported any signs of cybersickness after 20 minutes of VR HMD exposure time. They rated comfort (median 10/10) and movement ability (median 10/10) with the VR HMD as high. The VE was perceived as being really there by the majority (median 8/10), and the participants had a strong feeling of spatial presence in the VE (median 9.5/10). They enjoyed exploring the virtual world (median 10/10) and liked this new therapy approach (median 10/10). Therapists’ acceptance of the VR HMD was high (4/5). There were 5 patients that needed more support than usual, mainly for supervision, when moving around with the VR HMD. Otherwise, therapists felt that the VR HMD hardly affected their patients’ movement behavior (median 4.75/5), whereas it seemed to increase their level of therapy engagement (median 4/5) compared to conventional physiotherapy sessions.

**Conclusions:**

This study demonstrates the usability of an immersive VE delivered through a VR HMD to engage youths in the training of everyday walking activities. The participants’ and therapists’ positive ratings on user experience and acceptance further support the promising application of this technology as a future therapeutic tool in pediatric neurorehabilitation.

## Introduction

Pediatric neurorehabilitation strives to provide patients with the greatest possible degree of independence in everyday life [1]. Therapies aimed at increasing children’s independence require them to actively work on their limitations and push their physical limits, which takes weeks, months, or even years—depending on the nature and severity of their impairment. Active involvement, perseverance, and adherence to the therapy program are crucial ingredients for these therapies, which incorporate various motor learning principles, to be successful [2]. However, it is particularly challenging for children to maintain all these qualities throughout a lengthy rehabilitation stay.

In recent years, computer technology applications that create virtual environments (VEs) have emerged more and more in the field of rehabilitation [3]. By merging the physical and virtual worlds, VEs enable task-specific training and can provide an ecologically valid environment similar to the real world [4]. Thereby, they offer new options for therapies and outcome assessment [5] while ensuring the safety of the therapy setting [4]. In addition, VEs can help increase children’s motivation and therapy adherence during the rehabilitation process, which may result in more training repetitions [6,7]. Further advantages are the enriched environments, exercise gamification, possibility of task-specific training incorporating variations and real-time feedback on performance, and easily adjustable difficulty levels to account for children’s different motor abilities [4,8,9]. When compared to conventional therapy or controls, VE interventions on the upper extremities, postural control, and balance in children with cerebral palsy showed a strong effect in improving motor functions [8]. So far, VE interventions targeting gait function in children with neuromotor impairment were considerably less frequent, and consequently, evidence on their effectiveness remains limited [6-8,10]. However, evidence derived from adult populations is promising. VE interventions effectively improved balance, gait functions, and mobility in various groups of patients with neurological disorders [6,11,12].

VEs differ regarding the display device, level of immersion, and type of interaction and can be delivered by custom-built systems as well as affordable off-the-shelf options [4,8]. To date, gait interventions using VEs mainly rely on nonimmersive flat-screen VEs. Although these approaches increase the enjoyment, motivation, and adherence toward the therapy program in pediatric and adult patients [6,7], they offer only limited possibilities in terms of interaction, sensorimotor contingencies, and illusions [13]. In contrast to these 2D VEs, virtual reality (VR) head-mounted displays (HMDs) provide a stereoscopic 3D view in a completely simulated environment while blocking the views of the real surroundings and user’s body. As some of these devices are wireless, they offer optimal prerequisites for motivating and immersive training of walking activities [13].

VR HMDs have already been shown to be feasible for balance or gait training in the older population [14] or adult patients with a neurological disorder [15,16]. However, in pediatric patients, the evidence of the VR HMDs’ feasibility and acceptability is very limited and restricted to their use in static positions, mainly during sitting or lying [17-20]. We have previously examined and reported on the usability and acceptability of 2 different HMDs (1 VR HMD and 1 mixed reality HMD) in children undergoing inpatient neurorehabilitation [21]. Although our first results were promising regarding an application of mixed reality or VR HMDs while moving around, the wearing time was short, and the VE did not allow for interactions. In this study, we now aimed to investigate the usability, user experience, and acceptability of an immersive VE with different game applications experienced through a VR HMD to train everyday life walking activities in children and adolescents with neuromotor impairments.

## Methods

### Participants

Children and adolescents aged 6 to 18 years with a neuromotor impairment and undergoing inpatient or outpatient rehabilitation at the Swiss Children’s Rehab (SCR) of the University Children’s Hospital Zurich were eligible. We aimed to have a diverse group of participants in terms of age, diagnosis, mobility level, visual acuity level, and cognitive abilities to test the VR HMD in a heterogeneous group representing the patient composition at the SCR. Exclusion criteria were inability to follow verbal instructions, uncorrectable severe visual impairment, and a history of seizures or taking anticonvulsant medication. Written informed consent and assent was obtained from the legal representatives and participating children and adolescents.

### VE Specifications

**Figure 1:**
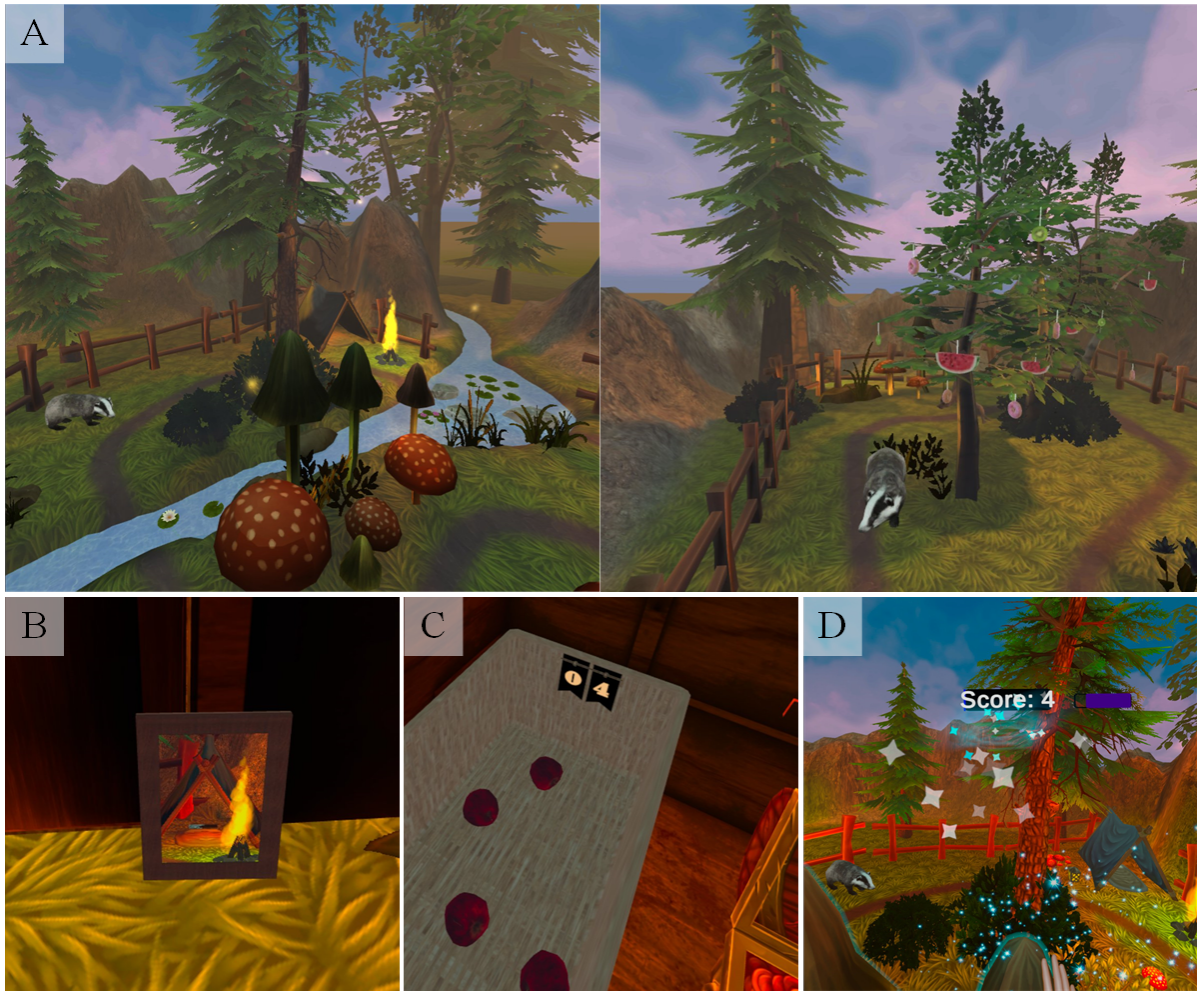
Virtual environment with (A) a magic forest, (B) the orientation game, (C) the apple game, and (D) the scoring game.

The VE, representing a magical forest (Figure 1), was created in the game engine Unity (version 2019.4.6; Unity Technologies). We chose the forest as the VE because it allowed integrating many everyday walking tasks considered important by parents and adolescents with neuromotor disorders [22]. Further, a forest represents a peaceful environment that allows the children to concentrate on tasks without being too distracted. Additionally, it can be appealing regardless of the user’s age and represents a situation that is not easily accessible in real life for many patients due to their functional impairments. Walking tasks that can be performed in the VE involved stepping over various obstacles such as roots, a tree log, stones, or a small creek; opening and closing the door to a hut; stepping over the door sill and moving around in the hut’s confined space; and picking sweets from a tree and carrying them to a badger to feed it. Further, the VE featured various mushrooms to play drums, lanterns to carry around, and a small tent that the children and adolescents could crawl into to grab a chocolate bar. The interactive objects should draw the participants’ focus to the inner walkable workspace and encourage them to walk around, whereas the normal objects such as hills and trees at the periphery should not attract their attention.

The participants experienced the VE through a commercially available VR HMD, the Oculus Quest 1 (Facebook Technologies). The Oculus Quest is a stand-alone device with 6 degrees of freedom and 4 integrated cameras, which enable room and hand tracking. The chosen visual design considered the performance of the Oculus Quest and the fact that a cartoon-like style is often appealing to children. The participants could freely move around the 4.4 by 10-meter VE (determined by the available space of our room and maximal given play zone of the Oculus Quest). We did not use the standard Oculus controllers as input devices, as holding or operating the controllers may have been difficult for some of our patients due to limited finger motor skills or reliance on a mobility aid. Therefore, we used the Oculus hand tracking that allows the participants to use their hands, which are displayed in a realistic and size-adapted manner in the VE, intuitively for interactions with the virtual objects. Possibilities for such interactions were placed throughout the VE and triggered auditory and visual feedback to motivate the participants to move around as much as possible.

The magical forest VE featured a *free exploration mode,* in which the children and adolescents could move around without specific instructions, and 3 different game conditions. In the *orientation game*, a picture frame revealed, upon being touched, an image of the location of the next picture frame, which the participant then had to find and touch, etc. In the *apple game*, the participants had to collect apples spread all over the VE and bring them to a basket in the hut. In the *scoring game*, the task was to score as many points as possible by interacting with the objects. Between interactions, participants had to cover at least 2 meters (indicated by a green bar at the top of the field of view) before they were able to score their next point.

A video of pediatric neurorehabilitation patients experiencing and interacting with the immersive virtual environment is shown in Multimedia Appendix 1.

### Ethical Considerations

The ethics committee of the Canton Zurich confirmed through a clarification of responsibility that approval for this cross-sectional study, which took place in the gait laboratory of the SCR, was not needed (Req-2020-00757).

### Protocol

After the participants were informed about the test procedure, the VR HMD was adjusted to their heads, and its optimal position was checked. The participants then started with the *free exploration mode*, which lasted 5 minutes. This mode allowed them to get used to the VR HMD and familiarize themselves with the VE and the interaction possibilities. Subsequently, the participants took off the VR HMD and answered short questions regarding potential symptoms of cybersickness, using the Virtual Reality Sickness Questionnaire (VRSQ) [23]. Thereafter, they played the 3 games described above, each lasting 5 minutes, in randomized order. Patients could play the 3 games without pausing or take off the VR HMD for a short break in between. Following the 3 games, the participants again answered the VRSQ and a short set of questions. A physiotherapist accompanied the patients to provide assistance if needed and observe their movement behavior and answered a questionnaire at the end of the session.

### Outcome Measures

Participant characteristics were retrieved from the patients’ medical records. Their functional level of mobility was rated by the physiotherapists with the Gillette Functional Assessment Questionnaire walking scale (FAQ) and the Functional Mobility Scale (FMS) [24]. The FAQ quantifies a range of walking abilities in daily life on an ordinal scale from 1 to 10, whereas the FMS complements the information by assessing the assistive device used over 5, 50, and 500 meters on an ordinal scale from 1 to 6.

Besides the VRSQ, the investigator used a customized questionnaire covering the aspects of comfort, fun, presence in the VE, and immersion to ask the participants about their experience with the VE and VR HMD. The questions mainly consisted of a subset of items from the Comfort Rating Scale [25], the Igroup Presence Questionnaire [26], and the Presence questionnaire 2.0 [27]. Further, the participants were asked how strongly they felt that the objects and environment they saw were really there. Our understanding was that if the environment and objects were experienced as actually being there, it would indicate an increased immersion and user experience. In turn, a positive user experience could result in an increased motivation of the patients to move around in the VE. The questions were answered by the participants on a visual analog scale. Further, the participants were asked to rank the 4 gaming conditions according to their preferences.

The physiotherapists’ questionnaire assessed the acceptability of the VR HMD as a therapeutic tool for their patient. Questions included their ratings of the participants’ movement behavior, level of support needed, and engagement during the VR session on a 5-point Likert scale. They were also asked about their opinion on using a VR HMD as a therapy tool and the advantages, disadvantages, and potential problems of using a VR HMD with their patients.

Furthermore, the absolute position of the VR HMD in the room was logged with a sampling rate of 50 Hz. Based on this data, the covered horizontal and vertical distance per gaming condition and participant was calculated.

### Statistical Analysis

Participants’ characteristics and covered horizontal and vertical distances are presented using descriptive statistics. Questionnaire responses are illustrated with frequencies, medians, and IQRs. To quantify potential differences between the 3 games, we tested the horizontal and vertical distances covered during the 3 gaming conditions for normal distribution and performed repeated measures ANOVA with Bonferroni-corrected post hoc tests. Calculated effect sizes were based on the *z* values of the nonparametric Wilcoxon rank sum tests. We interpreted *ω*^2^>0.01 as small, *ω*^2^>0.06 as moderate, and *ω*^2^>0.14 as large effect [28].

## Results

### Participant Characteristics

In total, 21 children and adolescents with a median age of 12.1 (IQR 5.5) years, of which two-thirds had a congenital neuromotor disorder, participated in this study (Table 1). The walking abilities of our study population were on a high level, as more than half (67%, n=14) of the participants were able to walk independently without an assistive device (FMS≥5), and 18 (86%) could walk outdoors at least for short distances (FAQ≥6). In all, 2 participants performed the VR HMD test in their wheelchair, 7 used a walking device, and 6 needed the help of their therapist, mainly in the form of supervision. No youth reported any signs of cybersickness in the VRSQ, neither after 5 nor 20 minutes of VR HMD exposure. Further patient characteristics are presented in Table 1.

Ratings on the usability of the VR HMD, user experience, and acceptability of the immersive VE are described in the following paragraphs.

**Table 1:** Descriptive characteristics of the study participants (N=21).

ID	Sex	Age (year)	Height (cm)	Diagnosis^a^	Glasses	FMS^b^	FAQ^c^	Mobility aid^d^
1	F^e^	8.2	120	Bilateral spastic-dystonic CP^f^ (II)	Yes	6, 6, 5	9	None
2	M^g^	12.3	152	Stroke	No	6, 6, 6	10	None
3	F	11.5	147	Bilateral spastic CP (III) with lower limb surgery	No	1, 1, 1	4	Wheelchair
4	M	15.2	167	Bilateral spastic CP (II)	No	5, 5, 5	8	None
5	M	9.0	121	Meningomyelocele with lower limb surgery	No	5, 2, 2	8	Posterior walker
6	F	9.4	147	Cerebral encephalopathy	No	6, 6, 6	9	None
7	M	17.7	183	Stroke	No	5, 5, 5	7	None
8	M	11.6	146	Traumatic brain injury	No	6, 6, 6	10	None
9	M	7.3	129	Stroke	No	3, 3, 3	8	Crutches
10	F	14.6	169	Traumatic brain injury	Yes	5, 5, NA^h^	6	None
11	F	10.6	127	Unclear, superimposed disease with spastic-dystonic gait disorder	Yes	5, 5, 5	9	None
12	M	6.8	114	Meningomyelocele with lower limb surgery	Yes	2, 1, 1	4	Posterior walker
13	M	12.1	150	Status post septic shock with ischemic cerebral lesions	Yes	5, 3, 2	7	None
14	M	9.1	140	Bilateral spastic CP (II) with lower limb surgery	Yes	2, 2, 1	7	Posterior walker
15	M	14.6	160	Meningomyelocele with lower limb surgery	Yes	2, 2, 1	6	Posterior walker
16	M	14.4	167	Bilateral spastic CP (III) with lower limb surgery	No	2, 1, 1	3	Wheelchair
17	F	16.0	168	Friedreich ataxia with spondylodesis T4-L3	No	2, 2, NA	6	Anterior walker
18	F	12.4	162	Meningomyelocele	Yes	6, 6, 6	10	None
19	F	15.5	161	Bilateral spastic CP (II) with lower limb surgery	No	5, 3, 3	7	Crutches
20	F	7.9	122	Unilateral spastic CP (I) with lower limb surgery	Yes	6, 6, 6	10	None
21	F	13.0	172	Congenital ataxia	No	5, 5, 5	9	None

^a^In children and adolescents diagnosed with cerebral palsy, the Gross Motor Classification System Level is given in parentheses.

^b^FMS: Functional Mobility Scale at 5, 50, and 500 m.

^c^FAQ: Gillette Functional Assessment Questionnaire walking scale.

^d^Mobility aid used during virtual reality head-mounted display testing.

^e^F: female.

^f^CP: cerebral palsy.

^g^M: male.

^h^NA: not assessed.

### Usability

The participants rated the comfort of the VR HMD after 20 minutes of exposure time almost exclusively as positive. Only 3 patients reported that the VR HMD caused uncomfortable pressure on the back of the head (ID 14) or nose (IDs 16 and 17). Only 1 participant (ID 17) stated that not seeing her own body while moving around was a problem. None of the participants regarded it as unfavorable that the VR HMD blocked their view of the natural environment. Consequently, the youths did not feel hindered in their movement abilities by the VR HMD, except for 2 patients (IDs 5 and 13), who reported that grasping and manipulating the virtual objects was somewhat difficult for them.

### User Experience

**Figure 2:**
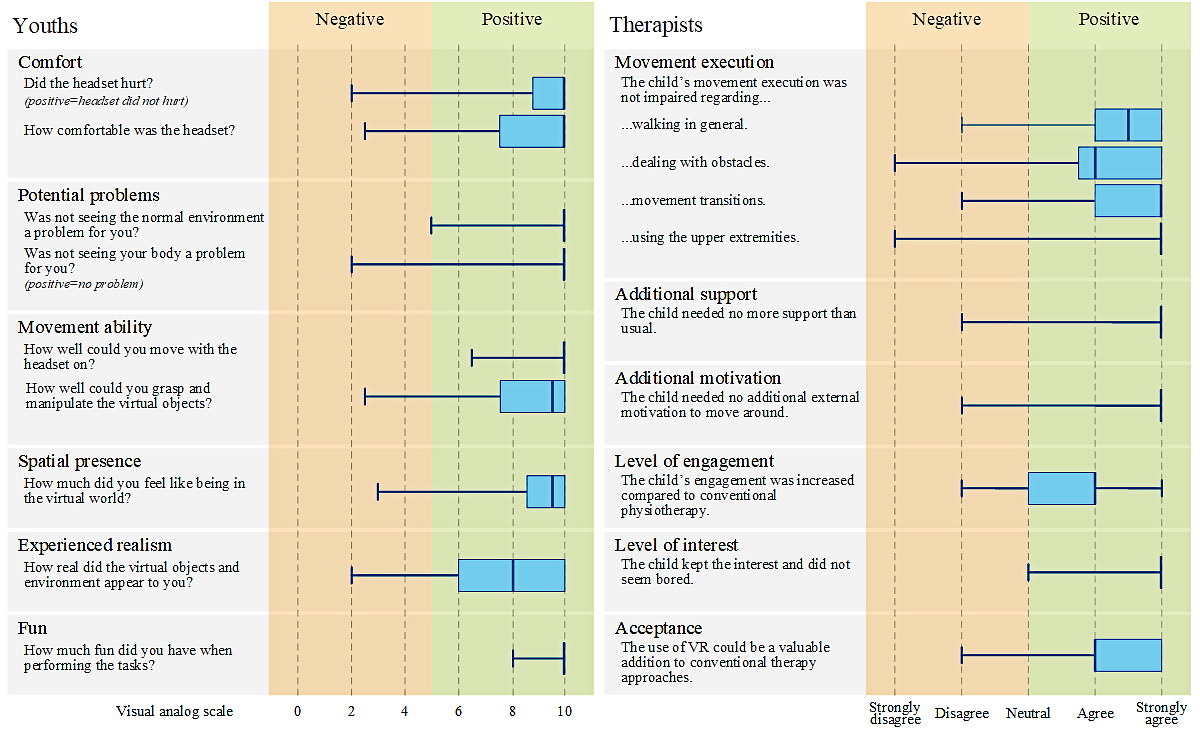
Youths’ and therapists’ ratings of various parameters regarding the usability of the virtual reality head-mounted display, the user experience, and the acceptability of the immersive virtual environment. VR: virtual reality.

The majority (90%, 19/21) of the participants felt that the VE and objects they saw were real (ie, really present). They also rated their feeling of being present in the VE as very high (median 9.5, IQR 1.5; Figure 2). When asked how much fun they had performing the tasks in the VE, 19 (90%) of the 21 patients gave the maximum score of 10. The badger and the possibility to feed it was most (71%, 15/21) participants’ favorite part in the VE.

### Acceptability

According to the physiotherapists, 5 patients needed more support than usual when moving around with the VR HMD, mainly in the form of supervision (IDs 7, 17, and 20) or assistance with their mobility aids (IDs 3 and 9). Otherwise, the VR HMD hardly affected the participants’ movement behavior. The physiotherapists rated their patients’ level of engagement during the VR session higher than in conventional physiotherapy (62%, 13/21; Figure 2).

They considered the immersive VE a valuable complement to conventional therapy methods for training everyday walking tasks in all but 3 participants (IDs 1, 12, and 21). Increased motivation, movement variations, repetitions, concentration level, playfulness, sense of achievement, joy of discovery, competition possibility with games, dual-task training, and reduced fear of movement were favorable factors mentioned by the physiotherapists. Potential problems or disadvantages identified by the therapists were the increased difficulty in handling the mobility aids when reaching for virtual objects, difficulty to work on gait quality, lack of haptic feedback, nonvisibility of the feet, weight of the VR HMD, and the VE not being challenging enough for some patients.

### VE Conditions

**Figure 3:**
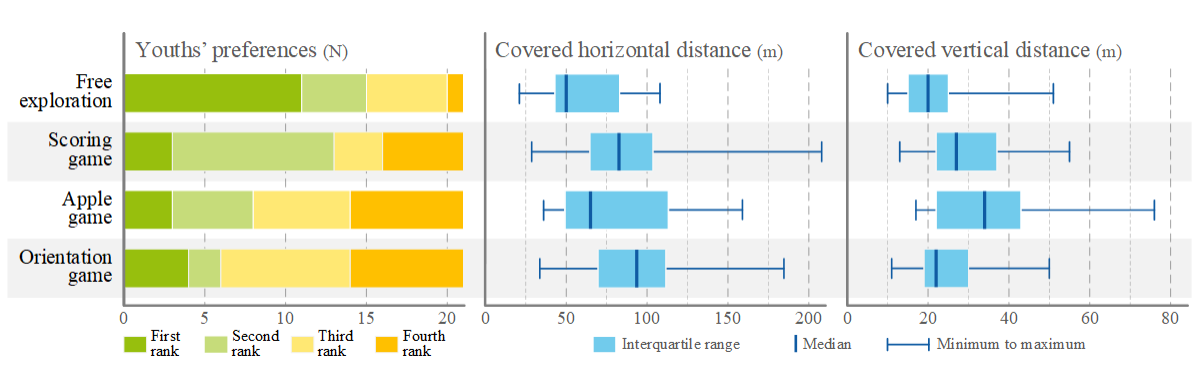
Overview of the 4 application modes by participant preferences and distance covered in the horizontal and vertical plane.

The *free exploration mode* received the best rating of the 4 conditions, followed by the *scoring game* (Figure 3). The participants covered the most horizontal distance in the *scoring game* (median 94 m; range 29-208 m). However, the repeated measures ANOVA showed no statistically significant difference between the 3 games (*F*_2,34_=2.60; *P*=.09).

The *apple collecting game* caused the largest covered vertical distance (median 34 m; range 17-76 m). The repeated measures ANOVA determined a statistically significant difference between the 3 games (*F*_2,34_=19.31; *P*<.001; *ω*^2^=0.5). Bonferroni-adjusted post hoc analyses revealed that the participants covered significantly more vertical distance in the *apple game* than the *scoring game* (*P*=.008) and *orientation game* (*P*<.001) as well as in the *scoring game* than the *orientation game* (*P*=.048).

## Discussion

### Principal Findings

We explored an immersive VE with different game conditions experienced through a VR HMD to train everyday life walking activities in a pediatric neurorehabilitation setting. The participants’ and therapists’ ratings regarding usability, user experience, and acceptability were very positive. The youths enjoyed moving around in the VE and experienced enormous fun—regardless of their age. Almost all participants felt comfortable with the VR HMD, and they did not notice any restrictions in their freedom of movement.

The participants reported a high sense of presence, indicating that they felt like being physically and spatially located in the VE. The sense of presence is a crucial feature of a VR application, as it directly influences users’ enjoyment during a VR game [29]. This high sense of presence might have also substantially contributed to the fact that the youths were not concerned by their blocked view of the natural surroundings. Remarkably, only 1 participant (ID17, diagnosed with Friedreich ataxia) considered the inability to see her body as problematic. The patients perceived moving around with the VR HMD as similar or equal to that in everyday life, but they rated their experience of activities involving hand and finger movements as slightly lower. Furthermore, 2 participants even experienced these activities as challenging, despite the visual representation of their hands in the VE. In fact, the integrated hand tracking option of the Oculus Quest caused some difficulties, as the hands are only detected as long as they are in the VR HMD cameras’ field of view. The partial concealment of the hand due to the patients’ hair or mobility aid and too fast hand and finger movements while grasping were other causes for the interrupted hand tracking of the device. Despite these challenges, the hand tracking feature enables an intuitive use of the hands in the VE, and the participants remained largely unperturbed and tried patiently to grasp the virtual objects.

From the physiotherapists’ point of view, the VR HMD hardly affected their patients’ movements, neither with respect to walking or crossing obstacles nor regarding movement transitions, using the upper extremities, or dealing with their walking devices. This finding is essential since a negative influence of the VR HMD on the patients’ movement execution would be unfavorable for efforts to validate its future clinical application. Furthermore, in contrast to our first study [21], therapists did not feel that their patients needed more support with the VR HMD than in standard therapy. This disparity in judgment might be because the therapists were already more familiar with this new technology and had more confidence that their patients could move safely even though they could not see the real environment.

Similar to the youths’ feedback, the therapists’ reported that their patients were more dedicated than during regular therapy sessions, for example, by being more interested and focused on the tasks or showing more perseverance. Further, they moved around without additional external motivation and did not seem to lose interest during the 20-minute test session. As motivation is an indispensable factor for active participation during the therapy, these findings are another indication of the promising option of immersive VR as a tool for movement therapy. Additionally, our results align with the findings of other studies that uniformly report the fun and enjoyment of various pediatric [17,18,20,21,30] and adult patient populations [15,16] when experiencing VR as a therapy instrument.

Despite the relatively high weight of the VR HMD, our patients could wear it for 20 minutes with almost no problems. Furthermore, we observed that the VR HMD is also suitable for different activities, such as walking, stooping, kneeling, crawling, or even running. This is new information, as in pediatric populations, the usability and acceptance of VR HMDs have so far only been tested in static situations [17-20] or when moving around for a short duration of a few minutes [21]. Studies with healthy adults and patients with stroke, multiple sclerosis, and Parkinson disease have demonstrated that VR HMDs could successfully be used while the participants were walking on a treadmill [15,16]. However, treadmill walking is much more restricted and controlled than moving around in an open space as our patients did. Everyday life activities usually happen in an unrestricted way and require a range of movement variations. Thus, our approach takes gait therapy even a step closer to daily life.

Whether a VR HMD is a helpful complement to conventional gait therapy methods depends on the patient’s individual abilities, preferences, and the specific therapy goals. Although the missing haptic feedback of not successfully mastering obstacles or the nonvisibility of the feet can make training on gait quality more difficult for some children and adolescents, this absence could help others increase their concentration on their proprioceptive input. Additionally, the possibility to train all activities on even ground might be helpful for some patients. However, the challenge could be too small for others because of an absent uneven terrain. According to the therapists, the patients often seemed to stay more concentrated for longer time periods and demonstrated more endurance and perseverance in the VE than in conventional therapy sessions. In the study of Lai et al [30], the participants reported that their exercise amount had been significantly facilitated by the immersive and enjoyable character of the VR HMD applications. The application’s novelty, the numerous discovery opportunities in the VE, and the games’ playfulness and competitive nature may, therefore, also have positively contributed to the positive findings in our study.

### Limitations and Future Considerations

The mobility level of our participants was at a high level, with 15 being community walkers with an FAQ level of at least 7. The comments of the physiotherapists, who considered our VE not to be the right approach to address some of these patients’ therapeutic goals, indicate that the VE was not challenging enough for patients who can master uneven grounds without any assistance (FAQ≥9), which applied to 8 participants of our study population. The introduction of various difficulty levels, dual-task training, and other VEs with more challenging tasks are possible future solutions to provide an adequate training level for these patients with higher functioning. As we did not compare the visual design of the current VE to others, we cannot comment on the effect of our particular visual design or VE choices.

Although we randomized the order of the 3 games, this was not the case for the *free exploration mode*. The participants always started with this condition as a type of familiarization with the VR HMD and VE. Therefore, we could not include the *free exploration mode* in the analysis regarding the covered distances. Neither can we rule out that the majority of the participants preferred the *free exploration mode*, because they experienced the VE with this condition in the first place. Nevertheless, it seems advisable to provide new VEs and interaction features always with an additional noncompetitive option, whether for familiarization or for children and adolescents who prefer noncompetitive games.

Our participant group was substantially heterogeneous in terms of age and motor abilities. Although it would have been interesting to analyze the impact of these characteristics, our sample size precluded forming any subgroups for further analyses. Additionally, as the study was cross-sectional, we cannot draw any conclusions about the impact of our VR HMD approach on the change in patients’ functional walking abilities or how their motivation would develop over the long term. Further, the assessment of the participants’ qualitative movement behavior was solely based on the subjective ratings of the youths themselves and their therapists.

Consequently, in a current study, we use 3D gait analysis to record spatiotemporal and kinematic parameters to compare the patients’ movements when performing similar activities in real environments and VEs. Furthermore, we aim to develop and implement a foot tracking option in the VE. This option would provide patients with visual feedback on their feet’s position and create further interaction possibilities, which would further help improve the VR experience. Last, the implementation of a movement therapy using a VR HMD is required in a clinical setting to evaluate its value and effect on patients’ motivation and movement skills.

### Conclusions

This study demonstrates the usability of an immersive VE delivered through a VR HMD in children and adolescents with neuromotor impairments performing everyday life walking activities. Furthermore, participants’ and therapists’ ratings regarding user experience and acceptability and the application’s high motivational impact support its development as a future tool for movement therapy in pediatric neurorehabilitation. However, in the light of the current generation’s rich gaming experience, choices, variation, difficulty levels, and other typical gaming features seem to be indispensable properties for successfully implementing the VR HMD as a therapy tool.

## Appendix

Multimedia Appendix 1 Video of pediatric neurorehabilitation patients experiencing and interacting with the immersive virtual environment.

## Abbreviations

FAQ: Gillette Functional Assessment Questionnaire walking scale
FMS: Functional Mobility Scale
HMD: head-mounted display
SCR: Swiss Children’s Rehab
VE: virtual environment
VR: virtual reality
VRSQ: Virtual Reality Sickness Questionnaire
